# Power-dependent spectral shift of photoluminescence from ensembles of silicon nanocrystals

**DOI:** 10.1186/1556-276X-7-389

**Published:** 2012-07-12

**Authors:** Dolf Timmerman, Tom Gregorkiewicz

**Affiliations:** 1 Van der Waals-Zeeman Institute, University of Amsterdam, Science Park 904, Amsterdam, NL-1098 XH, The Netherlands

**Keywords:** Nanocrystals, Silicon nanocrystal ensemble, Photoluminescence

## Abstract

Nanocrystals are widely studied for their tunable optical properties, most importantly increased luminescence efficiency and emission energy. Quantum confinement effects are found for many different types of nanocrystals, and these introduce a relation between the emission wavelength and the size of nanocrystals. When ensembles of nanocrystals with a distribution of sizes are studied, this can have profound effects on their luminescence spectra. Here, we show how photoluminescence spectra of ensembles of silicon nanocrystals can shift under different excitation conditions, resulting from differences in absorption cross-section of the individual nanocrystal sizes. This effect, together with the fact that after a pulsed excitation a silicon nanocrystal can only emit a single photon, determines how the distribution of excited nanocrystals changes and leads to the spectral shift for different excitation powers. Next to this effect, the influence of different radiative rates in such ensembles is also addressed. These notions are important for the interpretation of photoluminescence data for silicon nanocrystals but can be extended to any nanoparticle system comprising size-distributed ensembles.

## Background

Nanoparticles of many different semiconductor materials are intensively studied for their interesting optical properties. Quantum confinement in these nanostructures leads to strong altering of radiative rates and energy structure. In general, stronger confinement in smaller particles gives rise to shorter emission wavelengths[1]. Due to limitations in size selectivity on this scale, ensembles of nanoparticles always show a distribution of sizes and shapes. This results in inhomogeneously broadened photoluminescence (PL) spectra. For indirect band gap semiconductors, there is a special interest in the use of nanocrystals (NCs). In the bulk equivalent of these material, electron-hole radiative recombination has very low probability, since the difference in crystal momentum between the top of the valence band and bottom of conduction band can not be compensated by photon emission. In this case, optical transitions need to be accompanied by phonons, lowering their probability. For example, the radiative lifetime of excitons in bulk silicon is in the order of milliseconds. In Si NCs, the momentum conservation rule is relaxed, as *k* is not a good quantum number any more, and radiative phononless transitions become more probable. This leads to a substantial decrease of the radiative recombination time, which can be observed by photoluminescence lifetime measurements (from milliseconds to microseconds for the smallest Si NCs) [2,3].

PL spectroscopy is a powerful tool that is often used to determine specific properties of NCs. Most importantly, once a relationship has been determined between the size and the associated photon energy, it can be used as a relatively simple experimental way to determine sizes and distributions hereof.

In this work, we study PL spectra of silicon NCs and their change for different excitation fluences. A combination of size-dependent absorption cross-section and saturation effects in these NCs has a profound effect on the measured PL spectrum. We show simulations of this effect and compare them with experimental data. The effect of excitation conditions on the spectral profile will be discussed. Next to this, the influence of different PL lifetimes present in an ensemble of NCs is also addressed.

## Methods

A silicon-rich silicon oxide film with a thickness of 350 nm was deposited on a quartz substrate by a radio frequency co-sputtering technique. Subsequent annealing of the sample at a temperature of 1,150°C in nitrogen atmosphere for 30 min induced formation of Si NCs. The layer of Si NCs in amorphous SiO_2_ created in this way was characterized by an excess Si amount of 5 vol.% and contained NCs with an average diameter of approximately 3.5 nm.

PL experiments were performed under pulsed excitation provided by an optical parameter oscillator, pumped by the 3rd harmonic of a Nd:YAG laser, with a wavelength of 416 nm (3 eV) and pulse width of approximately 9 ns. Luminescence was dispersed by a monochromator (Solar M266, SOLAR Laser Systems, Minsk, Republic of Belarus) and detected by a linear detector (Hamamatsu S10141-1108s, Hamamatsu Photonics K.K., Shizuoka Pref., Japan). The entire system was calibrated for its spectral response with a calibration source. All measurements were taken at room temperature.

## Results

There is a lot of literature available on the dependence of the band gap of the Si NCs on the diameter. For an overview hereof, see [4]. For oxidized NCs, it is commonly reported that this dependence follows a *D*^−*b*^ law, where 1 <*b*< 1.5 and *D* is the NC diameter. The relation between emission energy and size of NCs determined by high-resolution transmission electron microscopy for the sample used in this study has been established earlier [5]. It was found that a good fit to the experimental data for the optical gap, *E*_*g*_, is given by the following equation:

(1) $$E_{g}(D)=E_{\mathrm{Si}}+\frac{1.86}{D^{1.39}},$$

where *E*_Si_ is the band gap of bulk Si, similar as the theoretical dependence determined in [6,7]. When an intrinsic yield of PL of 100% is assumed for all sizes [8,9], we can obtain a size distribution directly from PL spectrum, as the emission wavelength is related to the band gap. It is important to note that this is only valid for the optically active NCs in the entire distribution. Different effects can quench or enhance luminescence from Si NCs, which could introduce a size-dependent emission efficiency. For example, a single dangling bond on the surface will quench PL completely [10], and the chance of that will be strongly size-dependent. Other effects like exciton hopping [11,12] and non-radiative radiation via defects [13] can change the wavelength-dependent quantum efficiencies and, thus, the final emission spectrum. If such effects are quantified for a certain system, this could be used in order to get information about the entire distribution from the determined PL spectra. For the rest of the analysis done here, however, we assume that all NCs are optically active.

Rewriting Equation 1 gives the relation between diameter and wavelength:

(2) $$D(E_{g})={\left(\frac{1.86}{E_{g}-E_{\mathrm{Si}}}\right)}^{\frac{1}{1.39}}.$$

**Figure 1:**
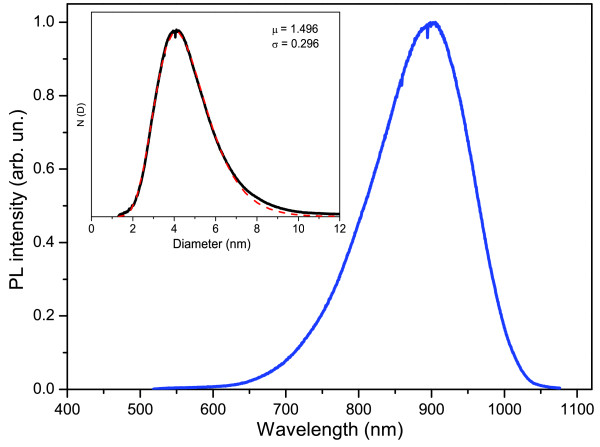
**Spectrum and size distribution.** PL spectrum obtained under high fluence excitation. Inset: The black line indicates the size distribution of the ensemble by converting the wavelengths from the measured PL spectrum with Equation 1. The dashed red line shows a fit of this distribution with Equation 3 with the fit parameters as shown in the figure.

The NCs prepared by the procedure used in this study commonly have sizes whose logarithm is normally distributed [14-16], described by Equation 3.

(3) $$N(D)=\frac{1}{\mathrm{D\sigma}\sqrt{2\Pi }}e^{-\frac{{\left(\ln(D)-\mu \right)}^{2}}{2\sigma^{2}}}$$

where *μ* and *σ* are the mean and standard deviation, respectively, for the natural logarithm of the diameter *D*. By fitting the determined size distribution with Equation 3, we can determine these important parameters. Figure 1 shows the result of this analysis, where values of *μ*and *σ* of 1.496 and 0.296, respectively, show a good fit with the experimentally determined PL spectrum. We note here that the fit actually deviates for larger NCs and underestimates its contribution. This is a result of the fixed limit that is imposed on the minimum optical gap (Equation 1), whereas in realistic systems, this will be less strict by participation of phonons in emission.

In reference [17], it was shown that, under the condition that the excitation pulse is much shorter than the PL lifetime, every NC can emit a single photon only. Multiple excitons localized in the same NC, that have been excited by multi-photon absorption, undergo a fast non-radiative Auger recombination. Finally, only a single exciton remains for radiative recombination. The number of excited NCs *N*^∗^ after an excitation pulse can, in this case, be described by Equation 4:

(4) $$N^{*}(J_{\mathrm{pump}})=N(1-e^{-\sigma_{\mathrm{abs}}J_{\mathrm{pump}}}),$$

where *N* is the number of NCs in the illuminated volume that can emit a photon, *σ*_abs_ the absorption cross-section, and *J*_pump_the pump fluence. From geometrical considerations, it is expected that the *σ*_abs_scales with the square of the diameter of the NC, which is theoretically supported by calculations of optical absorption cross-sections for Si NCs of different sizes [18]. We can now write $\sigma_{\mathrm{abs}}=\gamma D^{2}$, where *γ* is a proportionality factor. In this case, Equation 4 can be written as follows:

(5) $$N^{*}(J_{\mathrm{pump}})=N(1-e^{-\gamma D^{2}J_{\mathrm{pump}}}).$$

We can use now the expression for the distribution of NC sizes from Equation 3 and combine this with Equation 5 to arrive to an expression for the number of excited NCs as a function of excitation pump fluence and their associated band gap energy:

(6) $$N^{*}(E_{g},J_{\mathrm{pump}})=N(D(E_{g}))(1-e^{-\mathrm{\gamma D}{\left(E_{g}\right)}^{2}J_{\mathrm{pump}}}).$$

**Figure 2:**
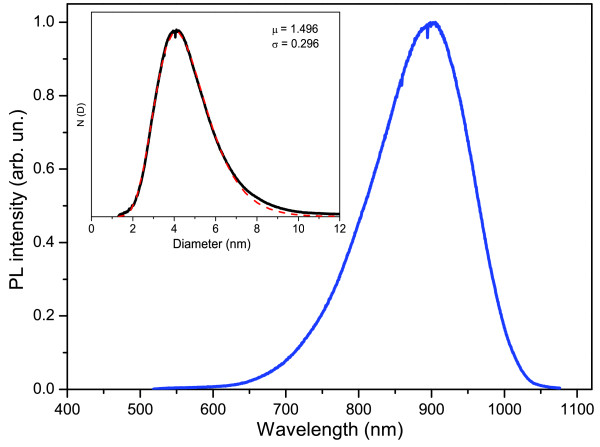
**Simulated power dependent spectra.** Spectra obtained from Equation 6 for pump fluence of 0.01 (red), 0.03 (orange), 0.07 (green), 0.3 (light blue), 0.65 (blue), and 1 (pink).

**Figure 3:**
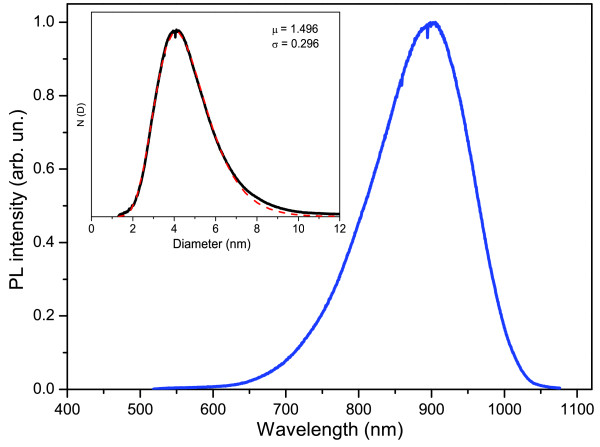
**Experimental and simulated power dependent spectra (normalized).** Left: Experimentally obtained normalized PL spectra for different excitation powers. The ratio of these powers is the same as the simulations in the left panel. Right: Simulated evolution of the normalized PL spectra for ratio’s of excitation power identical to that of Figure 2. Inset: Comparison of experimental and simulated peak wavelengths for the different excitation powers.

By converting *E*_*g*_ to wavelength with the relation $\lambda =\frac{\mathrm{hc}}{E_{g}}$, the associated PL spectrum can be obtained. Qualitatively, this equation describes how the ratio of excited NCs for different sizes changes upon illumination with different excitation powers. So, under assumption that the PL quantum efficiency is identical for all sizes, it shows how the PL spectrum changes with laser power. Larger NCs have a larger absorption cross-section, so their emission will be saturated at smaller pump fluences than for the smaller NCs. This means that, when the pump fluence is increased even more, the smaller NCs can still increase their contribution to the total (ensemble) emission. As a result, the PL spectrum will shift to shorter wavelengths. Figure 2 presents PL spectra obtained with Equation 6 for six different values of pump fluence of 0.01, 0.03, 0.07, 0.3, 0.65, and 1. While the intensity increases, saturation sets in at longer wavelengths, and the peak shifts to shorter wavelengths. A comparison of experimental data and the simulations is shown in Figure 3. We note here that carrier multiplication, known to occur in similar systems [19], has, in this case, been eliminated by using an excitation photon energy below the threshold of two times the NC band gap. For clarity, the normalized spectra are shown. In the simulations, the same ratios of excitation fluence are used as in the experiment. The value of *γ*was determined from the saturation behavior at a single wavelength by the method presented in references [17,20]. In this case, Equation 4 is fitted to the power-dependent PL intensity to obtain *σ*_abs_for a specific wavelength, which is subsequently used to deduce a value for *γ*. The simulation models the experimental spectra well; the peak shifts to shorter wavelengths, and the shift on the short wavelength side is more pronounced than that of longer wavelengths. The position of the peaks determined from the simulation and of the experiments shows a linear dependence. The small difference in the absolute numbers here is a result of the underestimation of contribution of larger NCs and, thus, longer wavelengths, which has been discussed earlier.

Next to a size-dependent absorption cross-section, there is also a dependence of the decay time [2]. Typically, smaller Si NCs have PL lifetimes in the order of 10 *μ*s, while for large NCs, this increases to a few hundreds *μ*s. This effect leads to red shift of PL spectra for longer detection times, which is often observed experimentally [21]. For Si NCs, most commonly a stretched exponential decay of PL intensity at a single wavelength is observed:

(7) $$I{\left(E_{g},t\right)}_{\mathrm{PL}}=I_{0}(E_{g})\cdot e^{-{\left(\frac{t}{\tau_{\mathrm{PL}}(E_{g})}\right)}^{\beta }},$$

where *β* is the stretch parameter; *τ*_*PL*_(*E*_*g*_), the emission energy-dependent decay time. In the regime where all NCs are excited, we can take the initial distribution described by Equation (3) and combine it with Equation (7) to arrive to an expression that describes the evolution of PL spectrum in time:

(8) $$I(E_{g},t)=N(E_{g})\cdot e^{-{\left(\frac{t}{\tau_{\mathrm{PL}}(E_{g})}\right)}^{\beta }}.$$

**Figure 4:**
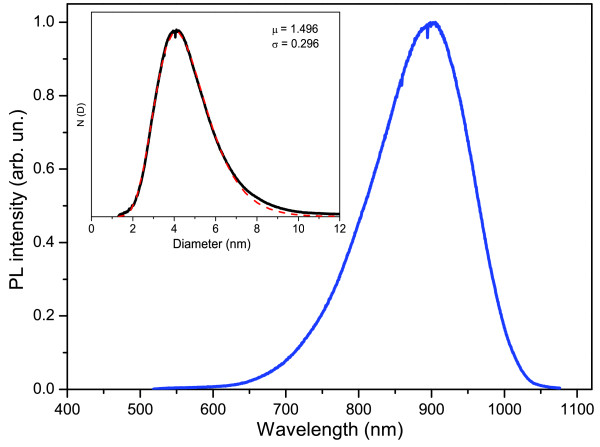
**Spectral evolution in time.** Simulated spectra for different times after a pulsed excitation of the entire ensemble of NCs, acquired from Equation 8. The spectral dependence of PL decay time, *τ*_*PL*_(*E*_*g*_), has been obtained experimentally. Left inset: PL intensity decay for detection wavelength of 800 nm. The red line indicates a fit with a stretched exponential function. Right inset: Experimentally determined PL lifetimes for different wavelengths (black squares). The red line shows an extrapolation for longer wavelengths.

For the wavelengths in the range of 600 to 850 nm, we have taken PL decays, and the traces have been fitted with the stretched exponential function to determine *τ*_*PL*_and *β*. It was found that *β* = 0.55 over the entire range, and the PL lifetime increases exponentially with detection wavelength in this region (see inset to Figure 4). For longer wavelengths, the *τ*_*PL*_was determined by extrapolation in order to be able to simulate spectra at different times after excitation. Figure 4 shows these results for *t* = 0, 10, 30, 50, 80, 100 and 150 *μ*s. A clear shift of the maximum PL intensity is accompanying the change of delay, very similar as observed in reference [21]. In the interpretation of such a time-dependent behavior, it is, thus, essential to remove contributions originating from the above nature.

## Discussion

The shift of PL spectra to shorter wavelengths when increasing the excitation power has been shown to occur in different types of NC ensembles. Different explanations have been suggested in order to account for this behavior [22-24], and the importance of the absorption cross-section has been noted as well [16,25]. Although the above proposed framework does not necessarily fully explain the experimentally observed shifts of PL spectra, it is important to account for the strong influence of the discussed effects. It should be emphasized that the analysis and simulations done here are based on the condition that the system is excited by a short pulse, with respect to the PL lifetime, and that the emerging luminescence is decaying fully in between the subsequent pulses. In experimental practice, this means that the pulse repetition rate should be small enough to allow all excited NCs to return to the ground state. Under continuous excitation, typical for the majority of experimental reports, the PL characteristics change considerably, as equilibrium conditions are important in this case. The size-dependent decay time could substantially alter the excitation power-dependent emission, and an even larger blueshift can be expected as the shorter PL lifetime of small NCs allows for more radiative recombinations. Now, the question arises under which experimental conditions PL spectra should be collected in order to get reliable information about the physical parameters of the system. For NCs showing long decay times, like Si, it is possible to excite the entire distribution by a pulsed excitation. After this, all multiple carriers simultaneously present in the same NC undergo a fast Auger non-radiative recombination, and every NC can, in principle, emit a photon. This would then be the best way to obtain information about the entire ensemble. However, high pump fluences might be necessary to arrive to this situation, and non-linear and heating effects can appear and influence PL properties [26]. Alternatively, at low powers where the average amount of absorbed photons per NC <<1, the shape of the PL spectrum will not change for varying power. This would be the best region to do comparative experiments, but the low intensity of PL signal might complicate the practical usability of this approach. It is also important to note that, in this case, PL will be dominated by contribution from large NCs. For more accurate information about smaller particles, a scaling based on absorption cross-section should be employed.

## Conclusions

To sum up, it has been shown that PL spectra of typical Si NC ensembles show a relatively strong dependence on the excitation fluence. A shift of 30 nm in the maximum of PL intensity appears between spectra taken at a low power excitation and under saturation conditions. It has been demonstrated that this shift can be modeled fairly well by taking into account the differences in absorption cross-section for different sizes of NCs. This, and the notion that each Si NC can only emit a single photon after a pulsed excitation, determines the distribution of the ensemble that is excited and contributes to PL. The evolution of the excited state distribution with respect to excitation pump fluence can be modeled fairly well by these simulations. Additional simulations, taking into account also differences in PL lifetime in the ensemble, show spectral narrowing and red shift for peak intensity in time-dependent spectra. While the presented modeling demonstrates the importance of excitation conditions on the PL spectra of ensembles of Si NCs, this approach can be extended to all semiconductor NCs, where ensembles and size-dependent absorption are imminent.

## Competing interests

The authors declare that they have no competing interests.

## Authors’ contributions

DT conceived the idea, developed theoretical descriptions and performed simulations. TG supervised the project. DT and TG co-wrote the paper. Both authors read and approved the final manuscript.

## Authors’ information

DT and TG are post-doctoral researcher and full professor, respectively, at the van der Waals - Zeeman Institute at the University of Amsterdam.
